# Real-World Effectiveness of Secukinumab in Congenital Ichthyoses: A Retrospective Monocentric Case Series

**DOI:** 10.3390/antib15040074

**Published:** 2026-08-11

**Authors:** Orsola Crespi, Umberto Santaniello, Luca Cangialosi, Ambra Bonvicino, Michela Ortoncelli, Francois Rosset, Luca Mastorino, Simone Ribero, Pietro Quaglino

**Affiliations:** 1 Dermatologic Clinic, Department of Medical Science, University of Turin, 10126 Turin, Italy; umberto.santaniello@unito.it (U.S.); lucacangialosi@unito.it (L.C.); abonvicino@cittadellasalute.to.it (A.B.); mortoncelli@cittadellasalute.to.it (M.O.); lucamastorino02@gmail.com (L.M.); simone.ribero@unito.it (S.R.); pietro.quaglino@unito.it (P.Q.); 2 Department of Dermatology, Beauregard Hospital, Azienda USL della Valle d’Aosta, Via L. Vaccari 5, 11100 Aosta, Italy; francois.rosset95@gmail.com

**Keywords:** congenital ichthyosis, lamellar ichthyosis, secukinumab, IL-17 inhibition, real-world evidence

## Abstract

**Background/Objectives:** Congenital ichthyoses (CI) are inherited disorders of keratinization characterized by epidermal barrier dysfunction, abnormal desquamation, and variable degrees of inflammation. Increasing evidence suggests a role for IL-23/Th17-mediated immune dysregulation in some CI subtypes, providing a rationale for targeted biologic therapies. Secukinumab, an anti-IL-17A monoclonal antibody, has emerged as a potential therapeutic option, although real-world evidence remains limited. This study evaluated the effectiveness of secukinumab in adult patients with severe CI. **Methods:** We performed a retrospective monocentric case series including eight adult patients with severe congenital ichthyosis treated with secukinumab for at least six months and followed for up to 56 months. Clinical outcomes included Investigator Global Assessment (IGA) scores for erythema, scaling, scalp involvement, and palmoplantar hyperkeratosis, together with pruritus assessed by Numeric Rating Scale (NRS). **Results:** Erythema and scaling showed most of their improvement during the first six months of treatment, after which mean clinical scores remained relatively stable throughout follow-up. At the last available assessment, improvement rates were 100% for erythema, 87.5% for trunk and limb scaling, 100% for scalp scaling, and 75% for palmoplantar hyperkeratosis. Hyperkeratosis showed a less pronounced response than inflammatory manifestations. Pruritus improved within the first 3–4 months of treatment and was sustained over time, with mean NRS scores decreasing from 6.5 at baseline to below 4 within 3–4 months. No discontinuations due to adverse events or lack of efficacy were recorded. **Conclusions:** Secukinumab was associated with clinically meaningful improvements in inflammatory manifestations of CI, particularly erythema and pruritus, while showing a more limited effect on hyperkeratotic features. These findings support the potential role of IL-17 inhibition in selected CI phenotypes.

## 1. Introduction

Congenital ichthyoses (CI) encompass a diverse group of rare inherited disorders characterized by abnormal epidermal differentiation, defective cornification, and chronic scaling of the skin. Traditionally, these conditions have been classified based on clinical presentation and underlying genetic mutations affecting proteins involved in lipid metabolism, keratinocyte differentiation, or epidermal barrier formation. However, the pathogenesis of CI extends beyond structural abnormalities and includes a significant inflammatory component that has only recently been elucidated.

Although congenital ichthyoses have traditionally been regarded as disorders primarily resulting from structural defects in epidermal differentiation and barrier formation, increasing evidence indicates that immune dysregulation also contributes to disease pathogenesis. Defective skin barrier function promotes chronic activation of innate and adaptive immune pathways, leading to sustained cutaneous inflammation and symptom burden. Transcriptomic and immunophenotypic studies have demonstrated increased expression of inflammatory mediators, particularly those associated with the IL-23/Th17 axis, across several forms of ichthyosis, including lamellar ichthyosis and other autosomal recessive congenital ichthyoses [1,2,3]. Elevated levels of IL-17-related cytokines have been associated with erythema, pruritus, and disease severity, suggesting that inflammatory mechanisms may represent relevant therapeutic targets in addition to the underlying genetic defects [1,2,3,4,5]. These findings have contributed to a paradigm shift in the understanding of congenital ichthyosis, supporting the development of targeted immunomodulatory approaches alongside conventional symptomatic treatments [5,6,7].

Seminal transcriptomic studies have demonstrated that major forms of CI share a common immunological signature dominated by IL-17-related pathways. In particular, Paller et al. showed that IL-17 signaling is markedly upregulated across several CI subtypes, including lamellar ichthyosis and congenital ichthyosiform erythroderma, suggesting a shared inflammatory axis despite genetic heterogeneity [1]. This observation has been reinforced by subsequent molecular analyses demonstrating profound Th17 polarization and dysregulation of barrier-related genes, indicating a bidirectional relationship between immune activation and epidermal dysfunction [2]. Furthermore, systemic immune involvement has been documented, with increased circulating Th17 and Tc17 cells and elevated inflammatory cytokines in peripheral blood, supporting the concept that CI are systemic inflammatory disorders rather than purely cutaneous diseases [3].

Additional studies have highlighted heterogeneity in the activation of the IL-23/Th17 axis among different CI subtypes, suggesting that the degree of inflammatory involvement may vary depending on the underlying genetic defect [4]. Nonetheless, the consistent presence of Th17-driven inflammation across multiple forms of ichthyosis has provided a strong rationale for targeting this pathway therapeutically [5].

The repositioning of biologic therapies originally developed for psoriasis and other inflammatory diseases represents a major advance in the management of CI. Secukinumab, a fully human monoclonal antibody that selectively inhibits IL-17A, has been widely used in psoriasis and has demonstrated rapid and sustained anti-inflammatory effects. Given the central role of IL-17 in CI pathogenesis, secukinumab has been proposed as a promising therapeutic option, particularly for patients with prominent inflammatory features such as erythema and pruritus [8,9].

A growing body of literature has explored the use of targeted therapies in CI, including biologics targeting the IL-17, IL-23, and IL-4/IL-13 pathways [10,11,12,13,14,15], as well as Janus kinase inhibitors such as upadacitinib [16]. Although several recent reports have shown promising results with these agents across different CI subtypes and related inherited keratinization disorders, the largest body of evidence currently concerns secukinumab, making IL-17A blockade the most extensively studied targeted therapeutic strategy in this field [14,15,17,18,19,20,21,22,23,24]. Early reports suggested that treatment leads to significant reductions in inflammation but only modest improvements in scaling, highlighting differential effects across clinical domains [21]. Subsequent real-world studies have confirmed that responses vary depending on disease subtype and phenotype, with better outcomes observed in inflammatory forms compared to hyperkeratotic variants [14]. Systematic reviews and expert guidelines have emphasized the potential role of biologics in CI while acknowledging the need for further evidence to define optimal patient selection and long-term efficacy [6,7,25,26].

In addition to lamellar ichthyosis, IL-17-targeted therapies have been investigated in a wide spectrum of inherited keratinization disorders, including epidermolytic ichthyosis, Netherton syndrome, and syndromic forms such as CHIME syndrome. These studies consistently demonstrate a reduction in inflammatory signs and symptoms, particularly pruritus, although the degree of improvement in scaling and hyperkeratosis remains variable [10,15,17,18,19,20,22,23,24,27,28,29,30,31]. The expanding use of biologics in these conditions reflects a paradigm shift toward mechanism-based therapy.

Despite these advances, data on long-term real-world outcomes of secukinumab in adult CI patients remain scarce. In particular, there is limited evidence regarding sustained efficacy, variability of response over time, and differential effects across clinical domains. The present study aims to address these gaps by evaluating the real-world effectiveness of secukinumab in a cohort of patients with severe congenital ichthyosis, with a focus on longitudinal clinical outcomes and comparison with existing literature.

## 2. Materials and Methods

### 2.1. Study Design

This retrospective, observational, monocentric case series was conducted at the Dermatologic Clinic of the University of Turin, a tertiary referral center for rare skin diseases. Adult patients with congenital ichthyosis were prospectively included in the institutional rare disease database and treated with secukinumab (Cosentyx^®^, Novartis Pharma AG, Basel, Switzerland) between April 2021 and March 2026 were retrospectively analyzed. Clinical and demographic data were extracted from medical records and systematically reviewed. The study was conducted in accordance with the principles of the Declaration of Helsinki. All patients provided written informed consent for treatment and for the use of anonymized clinical data for research purposes.

### 2.2. Patient Selection

Eligible patients were adults (≥18 years) with a diagnosis of congenital ichthyosis established by clinical evaluation. All diagnoses had previously been confirmed by genetic testing during routine diagnostic work-up; however, the original molecular reports, including the specific pathogenic variants, were not consistently available for retrospective review. Patients were included if they presented with moderate-to-severe disease requiring systemic treatment and had received secukinumab for at least six months. Patients with insufficient follow-up data or treatment duration shorter than six months were excluded from the analysis.

All patients had previously received conventional treatments, including topical emollients and keratolytics, while selected patients had been treated with systemic retinoids or phototherapy according to routine clinical practice.

### 2.3. Treatment Regimen

All patients received secukinumab at a dose of 300 mg administered subcutaneously every four weeks following an initial induction phase. Concomitant treatments, including topical therapies, systemic retinoids, and phototherapy, were permitted according to routine clinical practice.

### 2.4. Clinical Evaluation

Clinical outcomes were evaluated using Investigator Global Assessment (IGA) scores for erythema, trunk and limb scaling, scalp scaling, and palmoplantar hyperkeratosis. Each domain was assessed on a five-point ordinal scale ranging from 0 (absent) to 4 (severe disease). Pruritus severity was assessed using a Numeric Rating Scale (NRS) ranging from 0 (no pruritus) to 10 (worst imaginable pruritus).

All clinical assessments were performed during routine follow-up visits by the same experienced dermatologist responsible for the congenital ichthyosis referral clinic at our institution, thereby ensuring consistency in outcome assessment throughout the study.

No imputation of missing data was performed. Analyses were based on all available observations at each time point (available-case analysis). Consequently, the number of evaluable patients varied across follow-up visits and outcome measures according to data availability.

Assessments were performed at baseline and during routine follow-up visits. To facilitate longitudinal analysis, visits were grouped into predefined time windows: T1 (3–4 months), T2 (6–9 months), T3 (12 months), T4 (18–21 months), T5 (24 months), T6 (30 months), T7 (36 months), T8 (42 months), T9 (48 months), and T10 (52–56 months).

For each clinical domain, mean and median scores were calculated at every timepoint. Individual changes from baseline to the last available follow-up visit were also analyzed, together with the proportion of patients demonstrating clinical improvement in each outcome measure. Because follow-up duration varied among patients, the number of evaluable patients at each time point is reported in the corresponding figures.

### 2.5. Safety Assessment

Safety was assessed throughout treatment by routine clinical evaluations performed at each follow-up visit and by laboratory monitoring. Clinical assessments included the evaluation of adverse events potentially associated with IL-17 inhibition, including infections, mucocutaneous candidiasis, injection-site reactions, and other treatment-emergent adverse events. Laboratory monitoring included complete blood count, liver and renal function tests. Screening for hepatitis B virus (HBV), hepatitis C virus (HCV), human immunodeficiency virus (HIV), and latent tuberculosis infection using the QuantiFERON-TB Gold test (QIAGEN, Hilden, Germany) was performed before treatment initiation and repeated annually during follow-up according to routine clinical practice.

### 2.6. Statistical Analysis

Descriptive statistics were performed using Microsoft Excel for Microsoft 365 (Microsoft Corp., Redmond, WA, USA) to summarize baseline characteristics and clinical outcomes. Due to the small sample size, results were interpreted descriptively.

## 3. Results

### 3.1. Patients Characteristics

The study included eight patients, of whom seven were affected by lamellar ichthyosis and one by Chanarin–Dorfman syndrome. The cohort comprised six women and two men, with a median age of 38.5 years (range, 31–67). Concomitant topical therapies were used by four patients, while three patients received concomitant acitretin and one patient received concomitant phototherapy. The mean follow-up duration was 34.1 months, with a median follow-up of 39.5 months (range, 6–56), allowing evaluation of both short-term and long-term treatment outcomes. Detailed demographic and clinical characteristics of the study population are reported in Table 1.

Detailed longitudinal values for all clinical outcome measures are reported in Supplementary Table S1.

### 3.2. Efficacy

At baseline, disease severity was high across all evaluated domains. Mean IGA scores were 3.88 for erythema, 4.00 for trunk and limb scaling, 3.75 for scalp scaling, and 3.75 for palmoplantar hyperkeratosis. Pruritus burden was also substantial, with a mean NRS score of 7.13 and a median score of 6.5, confirming the severe clinical status of the study population prior to treatment initiation (Supplementary Table S1).

Compared with baseline, mean erythema scores decreased by 32.2% at T2 (6–9 months) and by 52.8% at T5 (24 months). Trunk and limb scaling improved by 37.5% at T2 and remained approximately 35–40% below baseline throughout most of the follow-up period. Scalp scaling showed the greatest improvement among the keratinization-related domains, decreasing by 43.2% at T2 and by 60.0% at T5. Palmoplantar hyperkeratosis demonstrated a slower response, with a reduction of 36.5% at T2 and 46.7% at T5.

**Figure 1:**
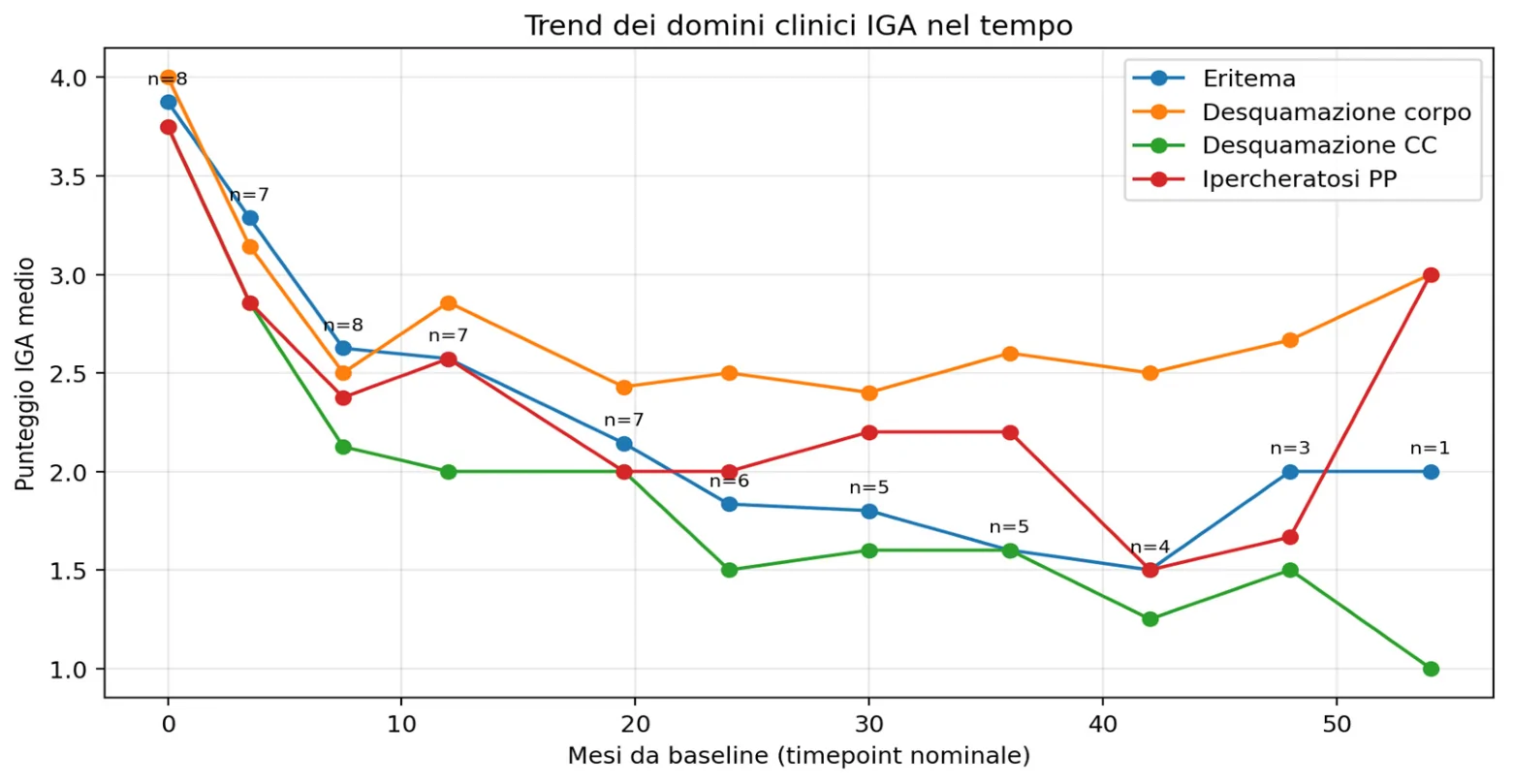
Longitudinal evolution of IGA scores over time. Numbers displayed next to each time point indicate the number of patients contributing data at that assessment. Most of the reduction in mean IGA scores occurred during the first six months of treatment, after which scores remained relatively stable, with greater variability at later follow-up time points.

Following the initial improvement phase, a plateau pattern was observed across most domains. Beyond 30 months of follow-up, greater variability emerged, likely reflecting both reduced patient numbers and heterogeneous long-term responses. Despite the plateau phase observed after the first year of treatment, clinical improvement was generally maintained throughout long-term follow-up. Mean erythema scores remained below 2.0 from month 24 onward, while scalp scaling remained consistently lower than baseline values across all subsequent assessments. Although fluctuations were observed at later timepoints, no evidence of complete loss of response was detected in any clinical domain (Figure 1).

At the last available follow-up, erythema improved in all patients, with individual reductions ranging from 1 to 3 points in IGA score, while scaling improved in the majority of cases. The proportions reported in Table 2 are based on the number of evaluable patients for each outcome measure at the last available follow-up. Owing to the retrospective collection of clinical data, scalp scaling and pruritus assessments were unavailable for one patient each, resulting in different denominators across outcomes. Improvement rates were 100% for erythema, 87.5% for trunk and limb scaling, 100% for scalp scaling, and 75% for palmoplantar hyperkeratosis. In contrast, palmoplantar hyperkeratosis showed a lower and less consistent response, with no measurable improvement observed in two patients, confirming a more limited impact of treatment on structural skin changes (Table 2).

Individual changes from baseline and responder rates are detailed in Supplementary Tables S2.

**Figure 2:**
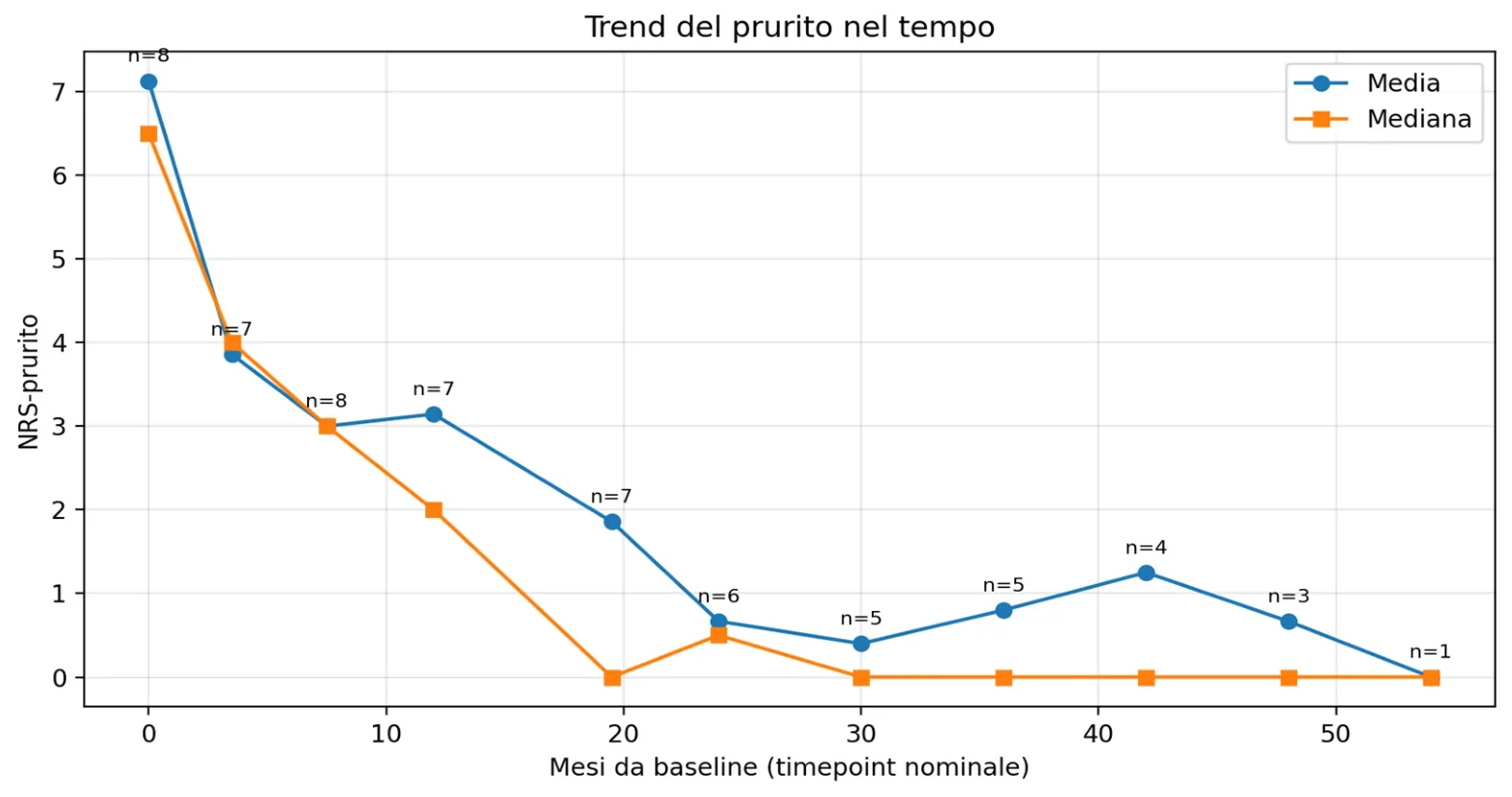
Evolution of pruritus (NRS) over time. Numbers displayed next to each time point indicate the number of patients contributing data at that assessment. Most of the reduction in mean NRS scores occurred during the first six months of treatment and was maintained throughout subsequent follow-up.

Pruritus demonstrated a distinct and particularly favorable trajectory, with a more pronounced improvement than any of the cutaneous clinical signs. A rapid and sustained reduction was observed throughout follow-up, with the mean NRS score decreasing from 7.13 at baseline to 3.86 at T1 (3–4 months) and remaining low thereafter (0.67 at T5, 24 months). Notably, the median pruritus score reached zero after approximately 20 months of treatment, indicating near-complete symptom control in most patients. Among all evaluated domains, pruritus showed the greatest magnitude of improvement, with reductions of up to 10 points on the NRS in individual patients. Among evaluable patients, reductions in pruritus ranged from 4 to 10 NRS points. Five of seven patients achieved reductions of at least 6 points, highlighting the substantial and clinically meaningful impact of secukinumab on symptom burden (Figure 2; Supplementary Tables S1 and S2). Overall, 85.7% of evaluable patients experienced an improvement in pruritus at the last available follow-up (Table 2).

Not all patients experienced uniform benefits across all disease domains. One patient showed no improvement in trunk and limb scaling or palmoplantar hyperkeratosis and experienced worsening of pruritus during follow-up. This finding further supports the heterogeneous nature of treatment response in congenital ichthyosis and highlights the need for biomarkers capable of predicting therapeutic outcomes.

### 3.3. Safety

Secukinumab was generally well tolerated throughout the observation period. Safety was assessed by clinical evaluation at each follow-up visit and by periodic laboratory monitoring, including complete blood count, liver and renal function tests, with annual screening for HBV, HCV, HIV, and latent tuberculosis.

No treatment-emergent adverse events were documented during follow-up. Specifically, no cases of upper respiratory tract infections, mucocutaneous candidiasis, injection-site reactions, inflammatory bowel disease, neutropenia, or clinically relevant laboratory abnormalities were observed. No serious adverse events related to secukinumab occurred, and no patient required temporary treatment interruption or permanent discontinuation because of adverse events.

Three patients discontinued treatment during follow-up for reasons unrelated to drug safety or lack of efficacy, namely pregnancy planning, breast cancer diagnosis, and personal reasons (Table 1).

## 4. Discussion

The present study provides real-world observations suggesting a potential clinical benefit of secukinumab in congenital ichthyosis, particularly in reducing inflammatory manifestations such as erythema and pruritus. These findings are consistent with the current understanding of CI as disorders involving both structural and immunological components, with previous studies suggesting an important role for the IL-17 pathway in disease pathogenesis.

The observation that most improvement in erythema and scaling occurred during the first six months of treatment is in line with previous reports demonstrating the efficacy of IL-17 inhibition in reducing cutaneous inflammation [14,21]. The relatively stable clinical scores observed thereafter suggest that, while inflammatory pathways can be effectively suppressed, residual disease activity may persist due to underlying structural abnormalities. This interpretation is supported by studies indicating that scaling and hyperkeratosis are less responsive to biologic therapy, reflecting their dependence on genetic defects in keratinization rather than immune-mediated processes [6,26]. However, methodological factors may also have contributed to this pattern. The IGA-based assessment used in this study may be more sensitive to changes in inflammatory features such as erythema than to more gradual changes in scaling and hyperkeratosis, potentially contributing to the smaller observed improvements in these domains. Future studies incorporating objective quantitative measures of scaling and skin thickness may help better characterize treatment effects on structural manifestations of congenital ichthyosis.

The limited response observed in palmoplantar hyperkeratosis is consistent with the hypothesis that structural manifestations of congenital ichthyosis are less responsive to IL-17 inhibition than inflammatory features. This observation is further supported by the individual patient-level analysis, which showed universal improvement in erythema but lower response rates for palmoplantar hyperkeratosis, suggesting that IL-17 blockade primarily affects inflammatory disease components while having a more limited impact on structural abnormalities of keratinization. Hyperkeratosis represents a hallmark of CI and is primarily driven by abnormalities in epidermal differentiation and lipid metabolism. Although inflammation may contribute to disease severity, it is unlikely to be the primary determinant of hyperkeratotic changes. Consequently, therapies targeting inflammatory pathways may have only a partial effect on these features.

The marked and sustained improvement in pruritus observed in our cohort highlights the potential contribution of IL-17 to sensory symptoms in congenital ichthyosis. As pruritus is a major determinant of disease burden, its sustained reduction may represent one of the most clinically meaningful benefits of IL-17 inhibition. Most of the improvement was observed during the first six months of treatment and was maintained thereafter, a pattern consistent with findings in other inflammatory skin diseases treated with IL-17 inhibitors. These observations are consistent with the hypothesis that IL-17 contributes to pruritus pathophysiology.

When compared with clinical trial data and other real-world studies, our findings confirm the heterogeneity of response to secukinumab in CI. Lefferdink et al. reported variable responses across CI subtypes, with better outcomes in patients with prominent inflammatory features [14]. Similarly, Subramani et al. observed significant reductions in inflammation but only modest improvements in scaling [21]. These observations are further supported by systematic reviews highlighting the differential effects of biologics on inflammatory versus structural disease components [26].

In lamellar ichthyosis, which constituted the majority of our cohort, recent case reports and small series have demonstrated variable responses to secukinumab, with improvements in erythema and pruritus but less consistent effects on scaling [15,17,24]. Pediatric studies have reported similar findings, suggesting that age may not significantly influence treatment response but that disease phenotype remains a key determinant.

The favorable safety profile observed in our cohort is consistent with previous clinical trials and post-marketing experience showing that IL-17 inhibition is generally well tolerated [32,33]. Although adverse events such as mucocutaneous candidiasis, upper respiratory tract infections, neutropenia, inflammatory bowel disease exacerbation, and injection-site reactions have been reported with secukinumab, none of these events occurred during the present study. This finding should nevertheless be interpreted cautiously given the limited sample size.

The variability observed in long-term outcomes in our study may reflect multiple factors, including disease heterogeneity, concomitant therapies, and potential adaptation of inflammatory pathways over time. Additionally, the progressive reduction in sample size at later time points limits the ability to draw definitive conclusions regarding long-term efficacy.

Overall, our findings suggest that patients with congenital ichthyosis characterized by prominent inflammatory manifestations may derive clinical benefit from IL-17 inhibition. However, given the retrospective and uncontrolled design of this study, these observations should be considered hypothesis-generating rather than confirmatory. Prospective multicenter controlled studies are needed to better define the efficacy of secukinumab, identify patients most likely to benefit, and evaluate long-term outcomes.

From a clinical perspective, these observations suggest that secukinumab may represent a promising therapeutic option for selected patients with predominantly inflammatory phenotypes who remain insufficiently controlled with conventional therapies. However, these findings should not be interpreted as evidence of treatment efficacy and require confirmation in prospective controlled studies.

The expanding use of biologics in congenital ichthyosis reflects a broader shift toward precision medicine in inherited disorders of keratinization. Future studies should focus on identifying biomarkers predictive of response to IL-17 inhibition and on further characterizing the immunological heterogeneity of different CI subtypes. In particular, the degree of IL-17/Th17 pathway activation, cytokine expression profiles, and transcriptomic signatures previously identified in congenital ichthyosis may help identify patients most likely to benefit from IL-17 blockade. Beyond predicting treatment response, these biomarkers could also improve disease stratification by distinguishing predominantly inflammatory phenotypes from those in which structural defects in keratinization are the principal drivers of disease. Such an approach may facilitate a more personalized therapeutic strategy, allowing biologic therapies to be preferentially offered to patients with a stronger inflammatory component while avoiding unnecessary treatment in those less likely to respond. Longitudinal molecular profiling before and during treatment may further improve our understanding of treatment response, resistance mechanisms, and the immunological changes associated with sustained clinical benefit. Given the rarity of these disorders, multicenter registries and international collaborative studies integrating clinical, molecular, and transcriptomic data will be essential to validate predictive biomarkers and generate more robust evidence regarding long-term efficacy and safety. Such integrated approaches may further support individualized therapeutic strategies and facilitate the development of mechanism-based treatment algorithms.

## 5. Conclusions

In this retrospective real-world case series, secukinumab was associated with clinically meaningful improvements in erythema and pruritus in patients with congenital ichthyosis, while more limited effects were observed for hyperkeratotic manifestations. Although these findings suggest a potential clinical benefit of IL-17 inhibition in selected inflammatory phenotypes, the uncontrolled design, small sample size, and clinical heterogeneity of the cohort preclude definitive conclusions regarding treatment efficacy. Larger prospective multicenter controlled studies are warranted to confirm these preliminary observations and to better define the role of secukinumab in congenital ichthyosis.

## 6. Limitations

This study has several limitations, including its retrospective, uncontrolled, single-center design, the small sample size, and the clinical heterogeneity of the study population. These factors limit the generalizability of our findings and preclude definitive conclusions regarding treatment effectiveness. An additional limitation is the absence of complete genotype information for all patients. Although all diagnoses had been previously established and genetically confirmed during routine clinical care, the original molecular reports were not consistently available for retrospective review, precluding genotype-specific analyses. Moreover, the limited sample size and the inclusion of only one patient with Chanarin–Dorfman syndrome did not allow meaningful comparisons according to disease subtype or genotype.

In addition, although concomitant therapies reflected routine clinical practice, the use of topical treatments, systemic retinoids, and phototherapy in some patients may have contributed to the observed clinical improvements and represents a potential source of confounding. The absence of a control group further limits the ability to distinguish the specific contribution of secukinumab from that of concomitant treatments or the natural variability of disease over time. Consequently, the observed improvements should be interpreted as associations rather than evidence of treatment efficacy or causality. Furthermore, although no treatment-related adverse events were observed, the limited sample size precludes definitive conclusions regarding the long-term safety of secukinumab in congenital ichthyosis. Finally, the reduced number of patients available at later follow-up time points limits the interpretation of long-term outcomes.

## Figures and Tables

**Table 1:** Baseline demographic and clinical characteristics of patients with congenital ichthyosis treated with secukinumab.

Patient	Age (Years)	Sex	Disease Subtype	Concomitant Topical Therapy	Concomitant Acitretin Therapy	Concomitant Phototherapy	Secukinumab Initiation Date	Total Follow-Up (Months)	Reason for Treatment Discontinuation	Last Follow-Up
1	67	Female	Lamellar ichthyosis	Yes	No	No	21 December 2022	25	–	4 February 2025
2	31	Female	Chanarin–Dorfman syndrome	Yes	No	No	16 February 2022	49	–	4 March 2026
3	35	Male	Lamellar ichthyosis	Yes	Yes	No	24 April 2021	56	–	30 December 2025
4	40	Female	Lamellar ichthyosis	Yes	No	Yes	6 July 2021	6	Pregnancy planning	7 January 2026
5	57	Female	Lamellar ichthyosis	No	Yes	No	7 December 2021	50	–	Ongoing
6	59	Female	Lamellar ichthyosis	No	No	No	7 December 2021	38	Breast cancer	11 April 2025
7	37	Male	Lamellar ichthyosis	No	No	No	9 April 2024	8	Personal reasons	1 September 2025
8	32	Female	Lamellar ichthyosis	No	Yes	No	26 July 2022	41	–	16 December 2025

**Table 2:** Proportion of patients showing improvement at the last available follow-up. The table summarizes the proportion of evaluable patients demonstrating improvement in each clinical outcome measure at their last available follow-up visit. Denominators vary according to the availability of outcome assessments.

**Outcome Measure**	**Patients Improved, n/N (%)**
Erythema (IGA)	8/8 (100.0%)
Trunk/Limb Scaling (IGA)	7/8 (87.5%)
Scalp Scaling (IGA)	7/7 (100.0%)
Palmoplantar Hyperkeratosis (IGA)	6/8 (75.0%)
Pruritus (NRS)	6/7 (85.7%)

Abbreviations: IGA, Investigator Global Assessment; NRS, Numeric Rating Scale.

## Data Availability

The data that support the findings of this study are available from the corresponding author upon reasonable request.

## Supplementary Materials

The following supporting information can be downloaded at https://www.mdpi.com/article/10.3390/antib15040074/s1, Supplementary Table S1. Longitudinal clinical outcomes during secukinumab treatment. Supplementary Table S2. Individual changes in clinical outcome measures from baseline to the last available follow-up.
