# Supplementary material for: Real-World Effectiveness of Secukinumab in Congenital Ichthyoses: A Retrospective Monocentric Case Series
Source: Antibodies (Basel). 2026 Aug 11;15(4):74. doi: 10.3390/antib15040074 (PMC13509879; doi:10.3390/antib15040074)
Supplement: Supplementary file 1 [file antibodies-15-00074-s001.zip › antibodies-4398800-supplementary.pdf]

Supplementary Table S1. Longitudinal clinical outcomes during secukinumab treatment.

The table reports the mean and median Investigator Global Assessment (IGA) scores for erythema, trunk and limb scaling, scalp scaling, and palmoplantar hyperkeratosis, together with pruritus severity assessed by Numeric Rating Scale (NRS), at each follow-up timepoint. N indicates the number of evaluable patients at each assessment.

Timepoint	N	Erythema Mean	Erythema Median	Trunk/Limb Scaling Mean	Trunk/Limb Scaling Median	Scalp Scaling Mean	Scalp Scaling Median	Palmoplantar Hyperkeratosis Mean	Palmoplantar Hyperkeratosis Median	Pruritus NRS Mean	Pruritus NRS Median
Baseline (T0)	8	3.88	4.0	4.00	4.0	3.75	4.0	3.75	4.0	7.13	6.5
T1 (3–4 months)	7	3.29	3.0	3.14	3.0	2.86	3.0	2.86	3.0	3.86	4.0
T2 (6–9 months)	8	2.63	3.0	2.50	2.5	2.13	2.0	2.38	2.0	3.00	3.0
T3 (12 months)	7	2.57	3.0	2.86	3.0	2.00	2.0	2.57	2.0	3.14	2.0
T4 (18–21 months)	7	2.14	3.0	2.43	2.0	2.00	2.0	2.00	2.0	1.86	0.0
T5 (24 months)	6	1.83	2.0	2.50	2.5	1.50	1.5	2.00	2.0	0.67	0.5
T6 (30 months)	5	1.80	2.0	2.40	2.0	1.60	2.0	2.20	2.0	0.40	0.0
T7 (36 months)	5	1.60	2.0	2.60	3.0	1.60	2.0	2.20	2.0	0.80	0.0
T8 (42 months)	4	1.50	1.5	2.50	2.5	1.25	1.0	1.50	2.0	1.25	0.0
T9 (48 months)	3	2.00	2.0	2.67	3.0	1.50	1.5	1.67	2.0	0.67	0.0
T10 (52 months)	1	2.00	2.0	3.00	3.0	1.00	1.0	3.00	3.0	0.00	0.0

Abbreviations: *IGA, Investigator Global Assessment; NRS, Numeric Rating Scale.*

Supplementary Table S2. Individual changes in clinical outcome measures from baseline to the last available follow-up.

The table reports the change (Δ) between baseline and the last available follow-up visit for each patient. Negative values indicate improvement in disease severity, whereas positive values indicate worsening. Missing values indicate unavailable assessments.

Patient	Last available timepoint	Last nominal month	Δ Erythema (IGA)	Δ Trunk/Limb Scaling (IGA)	Δ Scalp Scaling (IGA)	Δ Palmoplantar Hyperkeratosis (IGA)	Δ Pruritus (NRS)
1	T5	24.0	-2	-2	-3	-3	-4
2	T9	48.0	-3	-2	NA	-4	-7
3	T10	54.0	-2	-1	-3	-1	-6
4	T2	7.5	-1	-1	-1	-1	NA
5	T9	48.0	-1	-1	-2	-2	-10
6	T7	36.0	-2	-2	-2	-2	-8
7	T4	19.5	-1	0	-1	0	+3
8	T8	42.0	-3	-2	-1	0	-6

Abbreviations: *IGA, Investigator Global Assessment; NRS, Numeric Rating Scale; NA, not available.*
